# Seroepidemiology of pandemic influenza A (H1N1) 2009 virus infections in Pune, India

**DOI:** 10.1186/1471-2334-10-255

**Published:** 2010-08-25

**Authors:** Babasaheb V Tandale, Shailesh D Pawar, Yogesh K Gurav, Mandeep S Chadha, Santosh S Koratkar, Vijay N Shelke, Akhilesh C Mishra

**Affiliations:** 1 National Institute of Virology, 20-A, Dr. Ambedkar Road, Pune 411001, India

## Abstract

**Background:**

In India, Pune was one of the badly affected cities during the influenza A (H1N1) 2009 pandemic. We undertook serosurveys among the risk groups and general population to determine the extent of pandemic influenza A (H1N1) 2009 virus infections.

**Methods:**

Pre-pandemic sera from the archives, collected during January 2005 to March 2009, were assayed for the determination of baseline seropositivity. Serosurveys were undertaken among the risk groups such as hospital staff, general practitioners, school children and staff and general population between 15^th ^August and 11^th ^December 2009. In addition, the PCR-confirmed pandemic influenza A (H1N1) 2009 cases and their household contacts were also investigated. Haemagglutination-inhibition (HI) assays were performed using turkey red blood cells employing standard protocols. A titre of ≥1:40 was considered seropositive.

**Results:**

Only 2 (0.9%) of the 222 pre-pandemic sera were positive. The test-retest reliability of HI assay in 101 sera was 98% for pandemic H1N1, 93.1% for seasonal H1N1 and 94% for seasonal H3N2. The sera from 48 (73.8%) of 65 PCR-confirmed pandemic H1N1 cases in 2009 were positive. Seropositivity among general practitioners increased from 4.9% in August to 9.4% in November and 15.1% in December. Among hospital staff, seropositivity increased from 2.8% in August to 12% in November. Seropositivity among the schools increased from 2% in August to 10.7% in September. The seropositivity among students (25%) was higher than the school staff in September. In a general population survey in October 2009, seropositivity was higher in children (9.1%) than adults (4.3%). The 15-19 years age group showed the highest seropositivity of 20.3%. Seropositivity of seasonal H3N2 (55.3%) and H1N1 (26.4%) was higher than pandemic H1N1 (5.7%) (n = 2328). In households of 74 PCR-confirmed pandemic H1N1 cases, 25.6% contacts were seropositive. Almost 90% pandemic H1N1 infections were asymptomatic or mild. Considering a titre cut off of 1:10, seropositivity was 1.5-3 times as compared to 1:40.

**Conclusions:**

Pandemic influenza A (H1N1) 2009 virus infection was widespread in all sections of community. However, infection was significantly higher in school children and general practitioners. Hospital staff had the lowest infections suggesting the efficacy of infection-control measures.

## Background

The first pandemic influenza A (H1N1) 2009 case in India was reported in Hyderabad city on 16^th ^May 2009 [1]. Pune city reported the first pandemic influenza A (H1N1) 2009 case on 22^nd ^June 2009. The first pandemic death in Pune on 3^rd ^August 2009 caused panic in the general public. Subsequently, widespread transmission was reported in community [2].

The critical need of population-based serology has been advocated to determine the extent of infection and age-specific infection rates [3]. Wide geographical variations are expected in the incidence of infection in different populations. Therefore, large serosurveys covering different areas and age groups at different times are necessary to understand the extent of the infection in community. Further, seropositivity in population may provide appropriate denominator for pandemic severity estimates and the data for delineation of risk populations for priority in vaccination [4].

Several studies have been conducted to address the issues of cross-reactivity or pre-existing immunity using sera from the archives or collections from the routine diagnostic or screening programmes [5-10]. Some studies were done involving hospital staff [11], blood donors and patients without acute respiratory illness [12].

Pune is one of the cities in India reporting higher number of cases and deaths during this pandemic [2]. We report results of serosurveys undertaken in Pune in the risk groups, general population and household contacts of the PCR-confirmed cases. We also tried to detect the change in seroprevalence over time by resurveys in the selected risk groups.

## Methods

### Study area

Pune is the second largest urban agglomeration in Maharashtra state in India. Its population is about 3.76 million as per the 2001 Census. Pune has tropical climate with an average annual rainfall of 580.9 mm. Usually, June to September are the monsoon months. Incidence of seasonal influenza is higher in rainy and winter seasons though activity continues throughout the year. Seasonal influenza A (H3N2) was the most predominant strain in the year 2009 [2].

### Study design and sampling

For determining baseline seropositivity, anonymous left-over sera from the archives, referred for dengue diagnosis during January 2005- March 2009 were selected randomly and tested. PCR-confirmed pandemic influenza A (H1N1) 2009 cases were also sampled for serodiagnosis along with their household members for understanding the transmission. The study subjects volunteered and provided informed consents before depositing blood samples. An effort was made to broadly represent major divisions of Pune city for selecting the risk groups and the community clusters for the survey.

The present cross-sectional serological survey was undertaken between August 15 and December 11, 2009. Hospital staff, general practitioners and school children and staff were surveyed as the risk groups. Hospital staff from nine hospitals included doctors, nurses and other support staff who were actually involved in patient care activities like screening, sampling, diagnosis, isolation and critical care in the hospitals designated for pandemic flu patients. General practitioners were the medical practitioners from nine different areas of the city and worked mostly as family physicians in community and the first point of contact for pandemic flu patients. Hospital staff was resurveyed after nine weeks and general practitioners were resurveyed after 13 weeks. School staff from five schools was surveyed on 15th August 2009. In September, children and the staff of four schools were surveyed. We considered school children and staff as the risk groups due to the possibility of higher transmission in the schools. Children from the schools were selected from all the divisions reporting cases along with at least one unaffected division or class.

Office staff, railway commuters, slum-dwellers and general population were surveyed in September. Railway commuters travelling daily by the same local train were surveyed for studying the effect of crowding. Office staff was surveyed to understand the extent of infection in workplaces as an indirect measure of the population. In a general population survey in October 2009, cluster sampling was employed for selecting 20 localities spread over wide areas with the inclusion of slums proportionate to the size. In each selected locality, a house-to-house survey was done and all the family members were invited to participate in the study.

Informed consents were obtained from the adult volunteer participants and from the parents of children in schools and population. Administrative approvals were obtained from the health, municipal and school authorities and religious leaders. The study was reviewed and approved by the institutional ethical committee for research on human subjects at the National Institute of Virology, Pune, India and was considered exempt as the study was undertaken during the ongoing outbreak and was essential for guiding mitigation activities.

### Case definitions

Influenza-like illness was defined as the patient presenting with fever and either of sore throat or cough or both or a recent history of such symptoms [13]. A PCR-confirmed case of pandemic influenza A (H1N1) 2009 was defined as an influenza-like illness patient positive for viral RNA by reverse transcriptase polymerase chain reaction (RT-PCR).

### Data and specimen collection

Information about the subject's age, gender, area of residence, occupation, workplace, number of persons in the household, travel, exposure details, and symptoms of respiratory illness, duration of disease, medical consultation, treatment, hospitalisation and outcome was recorded. Blood samples (3-5 ml) were collected in evacuated tubes by venipuncture, kept at room temperatures for 30-45 minutes for allowing clot retraction and transported on wet ice within 4-6 hours of sampling. Sera were separated by centrifugation. Aliquots were made and stored at -20°C until testing.

### Laboratory procedures

All sera were treated with receptor destroying enzyme (Denka Seiken, Japan) for removal of non-specific inhibitors. In each assay, serum control included testing of serum without test antigen. Sera without non-specific agglutinins showed button formation, whereas sera with non-specific agglutinins showed haemagglutination. Sera with non-specific agglutinins were treated with turkey red blood cells, which removed non-specific agglutinins. The final dilution of the serum was 1:10. The pandemic influenza A (H1N1) 2009 virus was grown in 10-day-old specific-pathogen-free embryonated chicken eggs (Venky's India Limited, Pune), inactivated using beta-propiolactone and used as an antigen. The seasonal influenza A (H1N1) and A (H3N2) antigens were obtained from World Health Organization Collaborating Center for influenza (Centers for Disease Control and Prevention, Atlanta, USA) and were used in the assays. Haemagglutination-inhibition (HI) assay is routinely used for determination of the antibodies in sera [14]. HI assay was performed for detection of antibodies using 0.5% turkey red blood cells. Titres were reported as the reciprocal of the highest dilution showing complete inhibition. Two-fold dilutions of sera were made starting with 1:10 and the highest dilution of 1:1280. An HI antibody titre of 1:40 or more was considered seropositive as reported in most studies during the ongoing pandemic. HI assay was repeated for test-retest reliability. We also estimated the proportion of sera with antibody titre at or above the minimum detection limit of 1:10 against pandemic H1N1 virus.

### Statistical methods

Sample sizes were estimated considering the absolute precision of 5% at 95% confidence level. Seropositivity was reported as percentages with 95% confidence intervals. Age groups were categorized as 0-4, 5-9, 10-14, 15-19, 20-29, 30-39, 40-49, 50-59 and ≥60 years. An age ≥60 years was further divided as 60-69 and ≥70 years. We analyzed seropositivity in broad age groups like children (0-19 years), adults (20-59 years) and elderly (≥60 years). The age-specific seropositivity was calculated and adjusted to the population of Pune (2001 Census) by direct age-standardization method [15].

## Results

Age and sex distribution of the study subjects sampled during the pre-pandemic period and those sampled from different study groups at various time points during the pandemic period is presented in Table 1. Pre-pandemic sera (n = 222) were collected between January 2005 and March 2009. Among them, 64 (28.8%) were <20 years, 69 (31.1%) were aged 20-59 years and 85 (38.3%) were aged ≥60 years. HI antibody titre ≥1:40 was detected in sera of only 2 (0.9%) subjects, both aged ≥60 years. Considering the minimum detectable titre of 1:10, 6 (2.7%) sera were positive, of which 4 were aged ≥60 years and 2 were aged 40-59 years (data not shown).

**Table 1:** Age and sex distribution of subjects in different study groups at various time points

Study groups	Period of sampling	Age group	Total, n (%)	Males, n (%)	Females, n (%)
Pre-pandemic	January 2005 - March 2009	All	222*	115	102
		< 20	64 (28.8)	45 (39.1)	18 (17.6)
		20-39	22 (9.9)	16 (13.9)	6 (5.9)
		40-59	47 (21.2)	23 (20.0)	24 (23.5)
		≥60	85 (38.3)	31 (27.0)	54 (52.9)
		NA	4 (1.8)	0 (0.0)	0 (0.0)
Hospital staff	August 2009	All	495	276	219
		20-39	317 (64.0)	186 (67.4)	131 (59.8)
		40-59	173 (34.9)	88 (31.9)	85 (38.8)
		NA	5 (1.0)	2 (0.7)	3 (1.4)
	October 2009	All	524	250	274
		20-39	295 (56.3)	154 (61.6)	141 (51.5)
		40-59	225 (42.9)	94 (37.6)	131 (47.8)
		NA	4 (0.8)	2 (0.8)	2 (0.7)
	November 2009	All	385	183	202
		20-39	294 (76.4)	159 (86.9)	135 (66.8)
		40-59	86 (22.3)	23 (12.6)	63 (31.2)
		NA	5 (1.3)	1 (0.5)	4 (2.0)
General practitioners	August 2009	All	385	244	141
		20-39	148 (38.4)	85 (34.8)	63 (44.7)
		40-59	200 (51.9)	131 (53.7)	69 (48.9)
		≥60	33 (8.6)	26 (10.7)	7 (5.0)
		NA	4 (1.0)	2 (0.8)	2 (1.4)
	November 2009	All	278	213	65
		20-39	132 (47.5)	98 (46.0)	34 (52.3)
		40-59	123 (44.2)	98 (46.0)	25 (38.5)
		≥60	21 (7.6)	15 (7.0)	6 (9.2)
		NA	2 (0.7)	2 (0.9)	0 (0.0)
	December 2009	All	225	156	69
		20-39	167 (74.2)	111 (71.2)	56 (81.2)
		40-59	52 (23.1)	39 (25.0)	13 (18.8)
		≥60	6 (2.7)	6 (3.8)	0 (0.0)
School staff	August 2009	All	348	97	251
		20-39	152 (43.7)	34 (35.1)	118 (47.0)
		40-59	178 (51.1)	54 (55.7)	124 (49.4)
		≥60	6 (1.7)	4 (4.1)	2 (0.8)
		NA	12 (3.4)	5 (5.2)	7 (2.8)
	September 2009	All	177	27	150
		20-39	100 (56.5)	17 (63.0)	83 (55.3)
		40-59	67 (37.9)	7 (25.9)	60 (40.0)
		≥60	5 (2.8)	0 (0.0)	5 (3.3)
		NA	5 (2.8)	3 (11.1)	2 (1.3)
School children	September 2009	All (< 20)	2527	755	1772
Railway commuters	September 2009	All	225	150	75
		20-39	40 (17.8)	22 (14.7)	18 (24.0)
		40-59	49 (21.8)	37 (24.7)	12 (16.0)
		≥60	1 (0.4)	1 (0.7)	0 (0.0)
		NA	135 (60.0)	90 (60.0)	45 (60.0)
Office staff	September 2009	All	233	175	58
		20-39	152 (65.2)	104 (59.4)	48 (82.8)
		40-59	78 (33.5)	69 (39.4)	9 (15.5)
		NA	3 (1.3)	2 (1.1)	1 (1.7)
Slum-dwellers	September 2009	All	651	292	359
		< 20	252 (38.7)	139 (47.6)	113 (31.5)
		20-39	248 (38.1)	95 (32.5)	153 (42.6)
		40-59	96 (14.7)	33 (11.3)	63 (17.5)
		≥60	27 (4.1)	13 (4.5)	14 (3.9)
		NA	28 (4.3)	12 (4.1)	16 (4.5)
General population	October 2009	All	2520	1138	1382
		< 20	877 (34.8)	471 (41.4)	406 (29.4)
		20-39	885 (35.1)	335 (29.4)	550 (39.8)
		40-59	515 (20.4)	213 (18.7)	302 (21.9)
		≥60	226 (9.0)	107 (9.4)	119 (8.6)
		NA	17 (0.7)	12 (1.1)	5 (0.4)
PCR-confirmed cases	September - October 2009	All	65	30	35
		< 20	45 (69.2)	19 (63.3)	26 (74.3)
		20-39	14 (21.5)	7 (23.3)	7 (20.0)
		40-59	3 (4.6)	2 (6.7)	1 (2.9)
		NA	3 (4.6)	2 (6.7)	1 (2.9)
Household contacts	September-October 2009	All	195	84	111
		< 20	43 (22.1)	21 (25.0)	22 (19.8)
		20-39	75 (38.5)	34 (40.5)	41 (36.9)
		40-59	47 (24.1)	21 (25.0)	26 (23.4)
		≥60	19 (9.7)	5 (6.0)	14 (12.6)
		NA	11 (5.6)	3 (3.6)	8 (7.2)

* 5 subjects did not report their gender, of which 1 was aged <20 years and other 4 had not reported their age.

NA = not available, PCR = polymerase chain reaction

Sera from 48 (73.8%) of 65 PCR-confirmed pandemic influenza A (H1N1) 2009 cases were positive (Table 2). We treated 441 sera with turkey red blood cells. These sera were randomly distributed and were few as compared to the large number of sera processed in the study. The test-retest reliability of HI assay in 101 sera was 98% for pandemic H1N1, 93.1% for seasonal H1N1 and 94% for H3N2. The seropositivity (titre ≥1:40) in different study groups at various time points during the pandemic (Table 2) is described in the following sections.

**Table 2:** Seropositivity among different study groups at various time periods in the year 2009

Study groups	Month of 2009	No. sampled	HI titre ≥1:10		HI titre ≥1:40	
			
			**No**.	% (95% CI)	**No**.	% (95% CI)
Hospital staff	August	495	43	8.7 (6.2-11.2)	14	2.8 (1.4-4.3)
	October	524	40	7.6 (5.4-9.9)	25	4.8 (3.0-6.5)
	November	385	77	20.0 (16.0-23.9)	46	12.0 (8.7-15.1)
General practitioners	August	385	62	16.1 (12.4-19.8)	19	4.9 (2.8-7.0)
	November	278	67	24.1 (19.1-29.1)	26	9.4 (5.9-12.8)
	December	225	80	35.6 (29.3-41.8)	34	15.1 (10.4-19.8)
School staff	August	348	23	6.6 (3.9-9.2)	7	2.0 (0.5-3.5)
	September	177	46	26.0 (19.5-32.5)	19	10.7 (6.2-15.3)
School children	September	2527	800	31.7 (29.8-33.5)	631	25.0 (23.3-26.7)
Railway commuters	September	225	27	12.0 (7.8-16.3)	13	5.8 (2.7-8.8)
Office staff	September	233	25	10.7 (6.8-14.7)	8	3.4 (1.0-5.8)
Slum-dwellers	September	651	67	10.3 (8.0-12.6)	23	3.5 (2.1-5.0)
General population	October	2520	242	9.6 (8.4-10.8)	151	6.0 (5.1-6.9)
PCR-confirmed cases	September- October	65	53	81.5 (72.1-91.0)	48	73.8 (63.2-84.5)
Household contacts	September- October	195	70	35.8 (29.2-42.6)	50	25.6 (19.5-31.8)

CI = confidence interval, HI = haemagglutination-inhibition, PCR = polymerase chain reaction

### I. Seropositivity in risk groups

#### A. Hospital staff

In the last week of August, 2.8% of the 495 hospital staff was seropositive. Seropositivity was not significantly different among doctors (7, 2.9%), nurses (3, 3%) and other staff (4, 2.7%) (data not shown). In the last week of October, seropositivity was 4.8% and it was not significantly different from that in August. In November, seropositivity (12%) was significantly (p < 0.001) higher than at the first survey (Table 2). In all serum sets, seropositivity in the 20-39 years age group was not significantly different than in the 40-59 years age group (data not shown). Among 104 subjects found seronegative at the first survey in August, 6 (5.8%) became seropositive at resampling after 9 weeks indicating new infections during the period.

#### B. General practitioners

In the last week of August, 4.9% of the 385 general practitioners were seropositive. The seropositivity in November (9.4%) and in December (15.1%) (Table 2) was significantly (p < 0.001) higher than in August. Seropositivity in the 20-39 years age group (17/132, 12.9%) was significantly (p < 0.05) higher than in the 40-59 years age group (5/123, 4.1%) in the subjects sampled only in November (data not shown). Among 43 subjects found seronegative at the first survey in August, 5 (11.6%) became seropositive at resampling after 13 weeks indicating new infections during the period.

#### C. School children and staff

Seropositivity among school staff was 2% on 15^th ^August 2009 and it increased to 10.7% by the end of September (Table 2). In the schools with the reports of PCR-confirmed cases, 11.8% school staff was seropositive as compared to 4.2% in a school without PCR-confirmed case. Among 96 subjects found seronegative at the first survey in August, 4 (4.2%) became seropositive at resampling after 5 weeks indicating new infections during the period.

Influenza-like illness was not reported by any of these 4 seropositive subjects.

The overall seropositivity among school children (631/2527, 25%) was significantly (p < 0.001) higher than the school staff (19/177, 10.7%) sampled in September (Table 2). In the schools with the reports of PCR-confirmed cases, 26.1% students were seropositive as compared to 16.3% in the schools without PCR-confirmed cases. Further, the classes with the reports of PCR-confirmed cases showed higher seropositivity than other classes (data not shown). The lowest seropositivity of 7.4% was reported in <5 years age group. The 15-19 years age group showed the highest seropositivity (42.2%), followed by 10-14 years age group (26.7%) (Table 3). Among 631 seropositive subjects, 79 (12.5%) reported the recent history of influenza-like illness. The highest incidence of influenza-like illness (22.6%) was recorded in the students from 15-19 years age group. Seropositivity of 56% for seasonal H1N1 and 27.3% for seasonal H3N2 was noted in school children (data not shown).

**Table 3:** Seropositivity in different age groups among school children in September 2009

Age group (years)	No. sampled	HI titre ≥1:10		HI titre ≥1:40	
		
		**No**.	% (95% CI)	**No**.	% (95% CI)
< 5	149	12	8.1 (3.7-12.4)	11	7.4 (3.2-11.6)
5-9	821	203	24.7 (21.8-27.7)	164	20.0 (17.2-22.7)
10-14	1306	446	34.2 (31.6-36.7)	350	26.7 (24.3-29.2)
15-19	251	139	55.4 (49.2-61.5)	106	42.2 (36.1-48.3)
Total	2527	800	31.7 (29.8-33.5)	631	25.0 (23.3-26.7)

CI = confidence interval, HI = haemagglutination-inhibition

### II. Seropositivity in other groups

**Figure 1:**
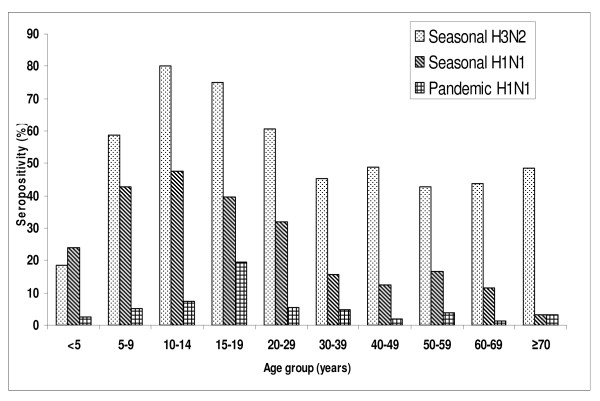
**Age-specific seropositivity of seasonal H3N2, seasonal H1N1 and pandemic H1N1 viruses in the general population in October 2009 (n = 2328)**.

In September, seropositivity was similar among railway commuters (13/225, 5.8%), office-staff (8/233, 3.4%) and slum-dwellers (23/651, 3.5%) (Table 2). Influenza-like illness was reported by 3 (13%) seropositive subjects from the slums. In a general population cluster survey of 2520 subjects in October 2009, the overall seropositivity was 6%. The seropositivity was similar in higher (8.3%), middle (5.2%) and lower (5.9%) social strata. Males and females had similar seropositivity. The highest seropositivity of 20.3% was observed in the age group 15-19 years (data not shown). A total of 192 subjects were excluded for presenting data in Fig. 1 because age was not recorded for 17 subjects and remaining 175 subjects had lacking data on seropositivity for seasonal H1N1 and H3N2 viruses. In 2328 subjects, seropositivity among children (73/790, 9.2%) was significantly (p < 0.001) higher than the adults (59/1538, 3.8%). Overall seropositivity of seasonal H1N1 and H3N2 in general population was 26.4% and 55.3% respectively. Whereas, among school-aged children sampled from community, seropositivity was 41.9% for seasonal H1N1 and 66.5% for seasonal H3N2. Seropositivity of H3N2 was significantly (p < 0.01) higher than seasonal H1N1 in all age groups except <5 years. Seropositivity of pandemic H1N1 (132/2328, 5.7%) was significantly (p < 0.01) lower than seasonal H3N2 in all age groups. Also, seropositivity of pandemic H1N1 was significantly lower than seasonal H1N1 in all age groups except in those aged ≥70 years (Fig. 1). The highest seropositivity of 19.6% for pandemic H1N1 was observed in 15-19 years age group followed by 7.2% in 10-14 years, 5.5% in 20-29 years and 4.7% in 30-39 years (Fig. 1). The lowest seropositivity of 1.3% was observed in 60-69 years age group. On direct age-standardization, age-adjusted seropositivity was similar to the age-specific seropositivity (data not shown).

### III. Household contacts of the PCR-confirmed cases

Among 195 household contacts of 74 PCR-confirmed cases, 50 (25.6%) were seropositive (Table 2). Among these, 7 (14%) reported influenza-like illness within 2-7 days of the onset of illness in the index case. The age-specific seropositivity was the highest in 5-19 years age group (40%), followed by 20-39 years age group (30.7%) (data not shown).

We further analyzed the proportions of subjects from different study groups whose sera were detected with the minimum detectable HI antibody titre of 1:10 (Table 2). Seroconversion in PCR-confirmed pandemic H1N1 cases was 81.5%. In the hospital staff, this proportion was 8.7% in August, 7.6% in October and 20% in November. Among general practitioners, these proportions were 16.1% in August, 24.1% in November and 35.6% in December. In school staff, positivity was 6.6% in August and 26% in September. School children had the highest positivity of 31.7% in September. In the schools, 55.4% of those aged 15-19 years and 34.2% of those aged 10-14 years were positive (Table 3). In other groups, these proportions were- 12% in railway commuters, 10.7% in office staff and 10.3% in slum-dwellers in September (Table 2). In general population, 9.6% subjects were positive, the highest of 28.4% in the age group of 15-19 years. In household contacts of PCR-confirmed cases, positivity was 35.8%. Overall, considering a titre cut off of 1:10, positivity was 1.5 - 3 times as compared to the titre cut off 1:40.

## Discussion

This is the first report of population-based large-scale serological surveys undertaken during the continuing influenza A (H1N1) 2009 pandemic in India. We also report the repeat surveys among adults in the selected risk groups for new infections over 5-13 weeks period and secondary infections among household contacts of the PCR-confirmed cases. We have surveyed a large number of school-aged children in the study. A recent report from New Zealand investigated 521 pre-pandemic sera and surveyed 1156 subjects (including 361 children) from community and 540 healthcare workers at the end of first wave [16]. Another study from Singapore investigated 727 adult community participants and 537 hospital staff [17]. A review of seroepidemiological studies clearly indicated the need of more studies using standardized methods to be able to accurately estimate global infection rate [18].

Seropositivity in the pre-pandemic period was negligible (0.9%) in our study. Other studies reported seropositivity in pre-pandemic sera at different levels [5-10,19]. A recent study from Singapore observed no or minimal cross-reactivity [17,20]. Indian population has experienced co-circulation of seasonal and pandemic viruses with almost equal contribution during the study period [2]. Also, seasonal influenza vaccination uptake is negligible. Seropositivity in the PCR-confirmed cases was 73.8% considering a cut off of 1:40 and 81.5% considering 1:10. Similar seroconversion of 89.1% in PCR-confirmed cases was reported using a titre cut-off of 1:32 in England [9].

The highest seropositivity was observed in the school children and the staff. Seropositivity was higher among students in the divisions with PCR-confirmed cases than in other divisions. Repeat serosurvey in school staff indicated significant increase in seropositivity after 5 weeks. The academic school calendar starts from June and continues through March. Seropositivity of 52% among 415 school children and staff in a residential school in Panchgani, India using a cut off titre of 1:10 [21] was higher than 31.7% in our study. Similar observations on incidence of influenza disease were reported during the school outbreaks in London [22] and New York [23]. In the UK, schools were identified as the most important source of infection [24]. Higher seropositivity in schools than households and communities could be attributed to the sustained close contact favouring transmission among the susceptible age groups [16].

In August, seropositivity among general practitioners (4.9%) was higher than the hospital staff (2.8%). During the repeat serosurvey, seropositivity was almost three times higher among general practitioners after 13 weeks. Among hospital staff, seropositivity was two times higher after 9 weeks. The higher seropositivity among general practitioners may be attributed to the higher possibility of contact with the patients as general practitioners are the first point of contact for influenza-like illness. Lack of infection-control practices may be another important reason. A study in Taiwan [11] reported 20% seropositivity among 295 hospital staff. Seropositivity of 29.6% in hospital staff and 25.3% in primary healthcare staff at the end of first wave in New Zealand indicated no additional risk to the healthcare workers as compared to general community (26.7%) [16]. The lower seropositivity among hospital staff in our study may be due to the widespread use of both therapeutic and prophylactic antiviral therapy and other infection-control measures.

In our study, higher seropositivity was noted in 15-19 years age group in both schools (42.2%) and in general population (20.3%). Seropositivity was lower in elderly population indicating low infection rates (Fig. 1). The incidence, severity and mortality of pandemic H1N1 disease was also lower in elderly than in other age groups in the study area [2]. The similar lower incidence of infection was reported in elderly in New Zealand [16]. This is also similar to the studies in some other countries [5-10]. It indicates that the pre-existing immunity and cross-reactivity levels vary in populations and age groups [20]. Seropositivity among elderly in China has been reported to be 9.4% in pre-pandemic and 42.5% in post-pandemic sera [25]. The likely hypotheses being forwarded include the differential exposures [26], the role of cell-mediated immunity [27] and immune epitopes or genetic differences [6].

Seropositivity of 25.6% in household contacts of the PCR-confirmed cases and influenza-like illness in 14% of those seropositive is noteworthy. The incidence of influenza-like illness among household contacts in our study was similar to the secondary attack rate of 13% detected in the USA [27]. The transmission of the pandemic H1N1 2009 virus is reported to be less-efficient in households as compared to the school settings [23]. It is also likely that the infections were mild, unnoticed or not remembered by the household members [23]. The widespread use of oseltamivir therapy in suspected cases and their household contacts, hospital or home isolation and the other prevention practices might have contributed to the lower infections in the household contacts. Post-exposure oseltamivir prophylaxis has been reported to reduce the rate of infection during outbreaks [28].

In our study, symptomatic infection was around 10%. Incidence of influenza-like illness among those identified with the serological infections in schools, hospital staff and general practitioners was similar to that reported in France [29], New Zealand [16,30] and the USA [31]. The 20-40% infections were reported to be symptomatic in the 1957 and 1968 pandemics [32,33]. The percentage of asymptomatically infected subjects was estimated at 2-10 times of the clinical cases during the current pandemic [9,34]. The asymptomatic infections seem to play an important role in transmission of the pandemic H1N1 virus as evidenced recently by RNA detection in persons with subclinical infection [35].

The possibility of underestimation of seropositivity may be due to the specificity and the threshold titre of HI assay [16]. This is evident from the data presented with a minimum detectable titre of 1:10 as compared to 1:40. The sera treated with turkey red blood cells were few and randomly distributed. We do not consider that these led to biases in the study. The pandemic H1N1 infections are widespread and mostly subclinical or mild. Asymptomatic or mild infections are reported to have low antibody titres as compared to clinically manifested patients [11]. There is under-representation of some age groups in general population. Information about underlying health conditions of 222 subjects sampled during pre-pandemic period could not be made available. Comparison of serology data with the incidence of clinical disease was not possible due to the non-availability of baseline seropositivity in representative pre-pandemic sera, non-reliability of disease incidence estimates due to changing policies of sampling and testing of throat swab specimens at different time periods, and co-circulation of seasonal and pandemic viruses in the community. We have certain limitations in estimation of incidence of infection as we could study only the adults by repeat surveys and limited number of household contacts of PCR-confirmed cases. Different levels of morbidity and mortality have been reported in different cities of India. Further serological surveys in different cities in India may help us understand infection rates and the factors responsible for such variation.

## Conclusions

We conclude that the schools played an important role for transmission of pandemic influenza A (H1N1) 2009 virus infections. General practitioners were at higher risk than the hospital staff. There is a need to provide health education regarding infection-control practices to these groups. The study may also help in prioritization for vaccination.

## Abbreviations

HI: Haemagglutination-inhibition; PCR: Polymerase chain reaction; RNA: Ribonucleic acid.

## Competing interests

The authors declare that they have no competing interests.

## Authors' contributions

BVT planned and designed field studies, executed and supervised field activities including collection of sera specimens, data collection and management, data analysis and interpretation, and wrote and revised the manuscript drafts. SDP standardized and supervised the assays, analyzed data and contributed in revising the manuscript drafts. YKG contributed in execution and supervision of field activities, and revising the manuscript drafts. MSC had significant inputs in planning, designing and guiding the field activities. SSK performed the assays and supervised the laboratory activities. VNS contributed in executing and supervising the field activities. ACM substantially contributed to the study design, guidance during field operations, interpretation of the results, reviewing and editing the manuscript drafts. All authors critically reviewed the drafts and approved the final manuscript.

## Pre-publication history

The pre-publication history for this paper can be accessed here:

http://www.biomedcentral.com/1471-2334/10/255/prepub

## References

[B1] Ministry of Health and Family Welfare, Government of India Update of influenza A (H1N1) as on 17th May, 2009 Press release dated 17th May, 2009 http://mohfw.nic.in/Status_as_on_16.5.2009_at_4.30_p.m.doc [Dated 15 Jun 2010]

[B2] Mishra AC Chadha MS Choudhary ML Potdar VA Pandemic influenza (H1N1) 2009 is associated with severe disease in India PLoS One 2010 5 5 e10540 10.1371/journal.pone.0010540 20479875 PMC2866330

[B3] World Health Organization Human infection with new influenza A (H1N1) virus: clinical observations from Mexico and other affected countries, May 2009 Wkly Epidemiol Rec 2009 84 185 189 19462531

[B4] Lipsitch M Riley S Ferguson NM Managing and reducing uncertainty in an emerging influenza pandemic N Engl J Med 2009 361 112 115 10.1056/NEJMp0904380 19474417 PMC3066026

[B5] Hancock K Veguilla V Lu X Zhong W Butler EN Sun H Liu F Dong L DeVos JR Gargiullo PM Brammer TL Cox NJ Tumpey TM Katz JM Cross-reactive antibody responses to the 2009 pandemic H1N1 influenza virus N Engl J Med 2009 361 1945 1952 10.1056/NEJMoa0906453 19745214

[B6] Xing Z Cardona CJ Preexisting immunity to pandemic (H1N1) Emerg Infect Dis 2009 15 1847 1849 19891882 10.3201/eid1511.090685 PMC2857244

[B7] Chen H Wang Y Liu W Zhang J Dong B Fan X de Jong MD Farrar J Riley S Smith GJ Guan Y Serologic survey of pandemic (H1N1) 2009 virus, Guangxi Province, China Emerg Infect Dis 2009 15 1849 1850 19891883 10.3201/eid1511.090868 PMC2857250

[B8] Ikonen N Strengell M Kinnunen L Osterlund P Pirhonen J Broman M Davidkin I Ziegler T Julkunen I High frequency of cross-reacting antibodies against 2009 pandemic influenza A(H1N1) virus among the elderly in Finland Euro surveill 2010 15 pii:19478 20144443

[B9] Miller E Hoschler K Hardelid P Stanford E Andrews N Zambon M Incidence of 2009 pandemic influenza A H1N1 infection in England: a cross-sectional serological study Lancet 2010 375 1100 1108 10.1016/S0140-6736(09)62126-7 20096450

[B10] Ross T Zimmer S Burke D Crevar C Carter D Stark J Giles B Zimmerman R Ostroff S Lee B Seroprevalence following the second wave of pandemic 2009 H1N1 influenza PLoS Curr Influenza 2010 RRN1148 20191082 10.1371/currents.RRN1148 PMC2828126

[B11] Chan YJ Lee CL Hwang SJ Fung CP Wang FD Yen DH Tsai CH Chen YM Lee SD Seroprevalence of antibodies to pandemic (H1N1) 2009 influenza virus among hospital staff in a medical center in Taiwan J Chin Med Assoc 2010 73 62 66 10.1016/S1726-4901(10)70003-4 20171584 PMC7129009

[B12] Allwinn R Geiler J Berger A Cinatl J Doerr HW Determination of serum antibodies against swine-origin influenza A virus H1N1/2009 by immunofluorescence, haemagglutination inhibition, and by neutralization tests: how is the prevalence rate of protecting antibodies in humans? Med Microbiol Immunol 2010 199 2 117 121 10.1007/s00430-010-0143-4 20162304

[B13] World Health Organization (WHO). Global influenza programme WHO manual on animal influenza diagnosis and surveillance, WHO/CDS/CSR/NCS http://www.who.int/vaccine_research/diseases/influenza/WHO_manual_on_animal-diagnosis_ and_surveillance_2002_5.pdf [Dated 15 Jun 2010]

[B14] Rowe T Abernathy RA Hu-Primmer J Thompson WW Lu X Lim W Fukuda K Cox NJ Katz JM Detection of antibody to Avian Influenza A (H5N1) virus in human serum by using a combination of serologic assays J Clin Microbiol 1999 37 937 943 10074505 10.1128/jcm.37.4.937-943.1999 PMC88628

[B15] World Health Organization (WHO) Age standardization of rates: A new WHO standard GPE Discussion paper series No. 31 http://who.int/healthinfo/paper31.pdf [Dated 15 Jun 2010]

[B16] Ministry of Health, New Zealand Seroprevalence of the 2009 influenza A (H1N1) pandemic in New Zealand http://www.nzdoctor.co.nz/media/224143/seroprevalence-flu-2009.pdf [Dated 15 Jun 2010]

[B17] Chen MIC Lee VJM Lim W-Y Barr IG Lin RTP Koh GCH Yap J Cui L Cook AR Laurie K Tan LWL Tan BH Loh J Shaw R Durrent C Chow VTK Kelso A Chia KS Leo YS 2009 influenza A(H1N1) seroconversion rates and risk factors among distinct adult cohorts in Singapore JAMA 2010 303 14 1383 1391 10.1001/jama.2010.404 20388894

[B18] World Health Organization Seroepidemiological studies of pandemic influenza A (H1N1) 2009 virus Wkly Epidemiol Rec 2010 85 229 236 20545056

[B19] Epstein SL Prior H1N1 influenza infection and susceptibility of Cleveland family study participants during the H2N2 pandemic of 1957: an experiment of nature J Infect Dis 2006 193 49 53 10.1086/498980 16323131

[B20] Tang JW Tambyah PA Wilder-Smith A Puong K-Y Shaw R Barr IG Chan K-P Cross-reactive antibodies to pandemic (H1N1) 2009 virus, Singapore Emerg Infect Dis 2010 16 5 874 876 20409391 10.3201/eid1605.091678 PMC2954004

[B21] Gurav YK Pawar SD Chadha MS Potdar VA Deshpande AS Koratkar SS Hosmani AH Mishra AC Pandemic influenza A (H1N1) 2009 outbreak in a residential school in Panchgani, Maharashtra, India Indian J Med Res 2010 132 67 71 20693592

[B22] Calatayud L Kurkela S Neave PE Brock A Perkins S Zuckerman M Sudhanva M Bermingham A Ellis J Pebody R Catchpole M Heathcock R Maguire H Pandemic (H1N1) 2009 virus outbreak in a school in London, April-May 2009: an observational study Epidemiol Infect 2010 138 183 191 10.1017/S0950268809991191 19925691

[B23] Lessler J Reich NG Cummings DA New York City Department of Health and Mental Hygiene Swine Influenza Investigation Team Nair HP Jordan HT Thompson N Outbreak of 2009 pandemic influenza A (H1N1) at a New York City school N Engl J Med 2009 361 2628 2636 10.1056/NEJMoa0906089 20042754

[B24] Ghani AC Baguelin M Griffin J Flasche S Pebody R van Hoek AJ Cauchemez S Hall IM Donnelly C Robertson C White MT Barrass I Fraser C Bermingham A Truscott J Ellis J Jenkins H Kafatos G Garske T Harris R McMenamin J Hawkins C Phin N Charlett A Zambon M Edmonds WJ Catchpole M Leach S White P Ferguson NM Cooper B The early transmission dynamics of H1N1 pdm influenza in the United Kingdom PLoS Curr Influenza 2009 RRN1130 20029668 10.1371/currents.RRN1130 PMC2780827

[B25] Jiang T Li X Liu W Yu M Liu J Yu X Qin E Cao W Leng Q Qin C Serum antibody response to the novel influenza A (H1N1) virus in the elderly Clin Infect Dis 2010 50 285 286 10.1086/649552 20034356

[B26] Chowell G Bertozzi SM Colchero MA Lopez-Gatell H Alpuche-Aranda C Hernandez M Miller MA Severe respiratory disease concurrent with the circulation of H1N1 influenza N Engl J Med 2009 361 674 679 10.1056/NEJMoa0904023 19564633

[B27] Cauchemez S Donnelly CA Reed C Ghani AC Fraser C Kent CK Finelli L Ferguson NM Household transmission of 2009 pandemic influenza A (H1N1) virus in the United States N Engl J Med 2009 361 2619 2627 10.1056/NEJMoa0905498 20042753 PMC3840270

[B28] Lee VJ Yap J Tay JK Barr I Gao Q Ho HJ Tan BH Kelly PM Tambyah PA Kelso A Chen MI Seroconversion and asymptomatic infections during oseltamivir prophylaxis against influenza A H1N1 2009 BMC Infect Dis 2010 in press 20537158 10.1186/1471-2334-10-164 PMC2901357

[B29] Flahault A de Lamballerie X Hanslik T Salez N Symptomatic infections less frequent with H1N1 pdm than with seasonal strains PLoS Curr Influenza 2009 RRN1140 20043034 10.1371/currents.RRN1140 PMC2798104

[B30] Baker MG Wilson N Huang QS Paine S Lopez L Bandaranayake D Tobias M Mason K Mackereth GF Jacobs M Thornley C Roberts S McArthur C Pandemic influenza A (H1N1)v in New Zealand: the experience from April to August 2009 Euro Surveill 2009 14 pii:19319 10.2807/ese.14.34.19319-en 19712648

[B31] Presanis AM De Angelis D New York City Swine Flu Investigation Team Hagy A Reed C Riley S Cooper BS Finelli L Biedrzycki P Lipsitch M The severity of pandemic H1N1 influenza in the United states, from April to July 2009: a Bayesian analysis PLoS Med 2009 6 e1000207 10.1371/journal.pmed.1000207 19997612 PMC2784967

[B32] Clarke SK Heath RB Sutton RN Stuart-Harris CH Serological studies with Asian strain of influenza A Lancet 1958 1 814 818 10.1016/S0140-6736(58)91739-2 13526280

[B33] Viboud C Grais RF Lafont BA Miller MA Simonsen L Multinational Influenza Seasonal Mortality Study Group Multinational impact of the 1968 Hong Kong influenza pandemic: evidence for a smoldering pandemic J Infect Dis 2005 192 233 248 10.1086/431150 15962218

[B34] Fraser C Donnelly CA Cauchemez S Hanage WP Van Kerkhove MD Hollingsworth TD Griffin J Baggaley RF Jenkins HE Lyons EJ Jombart T Hinsley WR Grassly NC Balloux F Ghani AC Ferguson NM Rambaut A Pybus OG Lopez-Gatell H Alpuche-Aranda CM Chapela IB Zavala EP Guevara DM Checchi F Garcia E Hugonnet S Roth C WHO Rapid Pandemic Assessment Collaboration Pandemic potential of a strain of influenza A (H1N1): early findings Science 2009 324 1557 1561 10.1126/science.1176062 19433588 PMC3735127

[B35] Yang J Yang F Huang F Wang J Jin Q Subclinical infection with the novel influenza A (H1N1) virus Clin Infect Dis 2009 49 1622 1623 10.1086/644775 19857173

